# A systematically collated library of prescribing safety indicators for people with chronic kidney disease

**DOI:** 10.1186/s12882-020-02158-0

**Published:** 2020-11-18

**Authors:** Fiona Smith, Samantha Hayward, Barnaby Hole, George Kimpton, Christine Sluman, Penny Whiting, Fergus Caskey

**Affiliations:** 1 North Bristol NHS Trust, Bristol, UK; 2 University of Bristol, Bristol, UK; 3 UK Renal Registry, Bristol, UK; 4 Applied Research Collaboration West, Bristol, UK

**Keywords:** Chronic kidney disease, CKD, Primary care population, Outpatient setting, Prescribing safety, Potentially inappropriate prescribing, Prescribing safety indicators

## Abstract

**Background:**

People with chronic kidney disease (CKD) have high levels of co-morbidity and polypharmacy placing them at increased risk of prescribing-related harm. Tools for assessing prescribing safety in the general population using prescribing safety indicators (PSIs) have been established. However, people with CKD pose different prescribing challenges to people without kidney disease. Therefore, PSIs designed for use in the general population may not include all PSIs relevant to a CKD population.

The aim of this study was to systematically collate a library of PSIs relevant to people with CKD.

**Methods:**

A systematic literature search identified papers reporting PSIs. CKD-specific PSIs were extracted and categorised by Anatomical Therapeutic Chemical (ATC) classification codes. Duplicate PSIs were removed to create a final list of CKD-specific PSIs.

**Results:**

Nine thousand, eight hundred fifty-two papers were identified by the systematic literature search, of which 511 proceeded to full text screening and 196 papers were identified as reporting PSIs. Following categorisation by ATC code and duplicate removal, 841 unique PSIs formed the final set of CKD-specific PSIs. The five ATC drug classes containing the largest proportion of CKD-specific PSIs were: Cardiovascular system (26%); Nervous system (13.4%); Blood and blood forming organs (12.4%); Alimentary and metabolism (12%); and Anti-infectives for systemic use (11.3%).

**Conclusion:**

CKD-specific PSIs could be used alone or alongside general PSIs to assess the safety and quality of prescribing within a CKD population.

**Supplementary Information:**

The online version contains supplementary material available at 10.1186/s12882-020-02158-0.

## Background

People with chronic kidney disease (CKD) stand to benefit from careful prescribing of medications to prevent disease progression, manage comorbidities and relieve symptoms [1]. Meanwhile they are at increased risk of prescribing-related harm as a result of incorrect dosing considering altered drug clearance or direct nephrotoxicity. Adverse drug events are common in the CKD population and interventions to optimise prescribing have shown promise [2]. However, current approaches for optimising prescribing in this patient group are limited [1–3].

Prescribing safety indicators (PSIs) distinguish prescribing events that put a patient at risk of harm [4]. PSI libraries for different patient populations have been collated through expert consensus, review of clinical guidelines, and systematic searching of published literature. One systematic approach to the identification of PSIs conducted by Spencer et al. in 2012 collated 56 indicators relevant to the general primary care population [4]. Whilst such a library is applicable to a CKD population, prescribing events of unique importance to people with kidney disease may be inadequately represented when considering pharmacotherapy for this group in isolation.

The aim of this study was to use a systematic search of the published literature to produce a library of PSIs specific to outpatient prescribing for people with CKD. This library could be used alone or alongside general PSIs to assess the safety and quality of prescribing within a CKD population.

## Methods

A protocol was written to collate PSIs with specific relevance to people with CKD from the published literature (PROSPERO CRD42018109113). A systematic literature search to identify papers reporting PSIs was followed by extraction of CKD-specific PSIs. Pre-defined criteria classified PSIs as CKD-specific if they were of exclusive relevance to adults (age ≥ 18) with CKD and referring to medications prescribed within the outpatient setting.

### Systematic literature search

A sensitive search strategy was designed to capture all potentially relevant papers reporting PSIs. To capture PSIs from the general primary care population the search strategy used by Spencer et al. was updated to include literature published from 2012 until 2018 [4]. Search terms related to three stems – clinical setting, prescribing and tool type. To reflect hospital-based delivery of advanced CKD care, a supplementary CKD-specific search was developed with revision of the clinical setting stem terms, and the addition of a stem for kidney-disease. CKD terms were chosen to capture all stages of CKD, including end-stage. This supplementary search was run from inception (1946 to 2018). Both searches were conducted in October 2018 using Medline, Embase, Pubmed, Web of Science and CINAHL. A full list of terms used in each search is available in Additional file 1: Appendix 1 (Table 3, 4).

Results from the two searches were combined and duplicate results were removed. Titles and abstracts were independently screened by two of the authors (FS, GK). An inclusive approach was used, excluding only those records from non-human or paediatric work (age < 18 years) or those referring solely to prescribing in an inpatient setting. There was no selection for CKD-specific PSIs made at this stage. If a record was recommended for inclusion by one of the reviewers it progressed to full text screening.

### PSI extraction and selection of CKD-specific PSIs

Full-text screening was conducted by FS. Publications reporting one or more PSI in the main text, abstract or tables of the publication were included. The details of all PSIs from the literature search were entered into a data extraction form by FS. The elements extracted included details relating to the publication itself – title; authors; journal; date of publication; study design; setting; target population; methodology and method of indicator development – in addition to exact replication of each PSI from the original publication.

A two-stage process followed to identify CKD-specific PSIs. Firstly, an automated filter was developed to shortlist possible CKD-specific PSIs. The automated CKD-specific filter terms (Additional file 1: Appendix 2: Table 5) were adapted and iteratively developed from the original search terms, through repeated comparisons between the automated process and manual review of a random sample of PSIs from the master list (BH). The final filter terms were selected once no manually included PSI was excluded by the automated process in a random sample of 100 PSIs. The output from this automated process was then divided between three authors (FS, BH, SH) who manually selected CKD-specific PSIs.

### Classification and categorisation of PSIs

Each CKD-specific PSI was labelled with the Anatomical Therapeutic Chemical (ATC) codes of each medication listed in the prescribing statement [5]. Duplicate PSIs and those not meeting pre-specified criteria were manually removed. In order to be classified as duplicates, two PSIs had to replicate the following information: identical drug, identical level of kidney function and identical prescribing rule. If a PSI was found to contain multiple rules, with other PSIs duplicating individual rules, then only the PSI containing the most information was maintained. Following duplicate removal, it was possible to identify the proportion of PSIs defined by their ATC classification and subsequently further divide PSIs in relation to the most frequent medication classes.

## Results

**Fig. 1:**
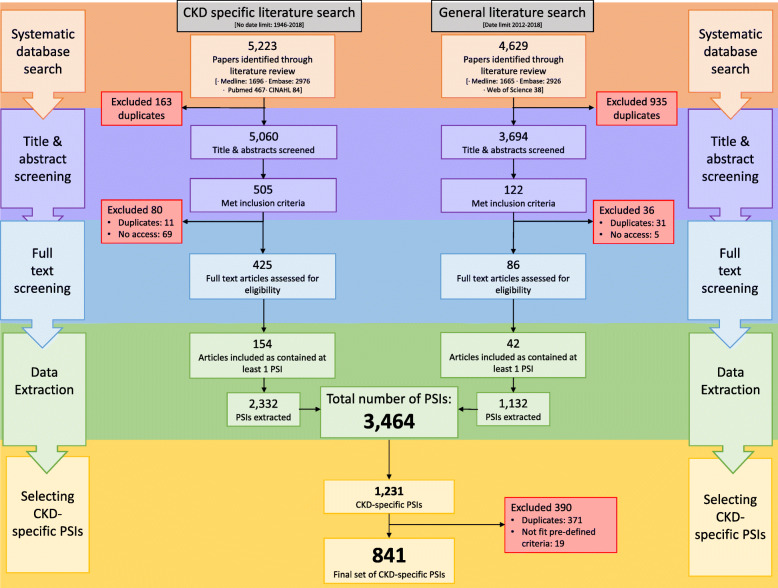
Systematic review results and PSI extraction

In total 9852 papers were identified by the two searches (CKD search 5223; general population search 4629). Following title and abstract screening 511 papers proceeded to full text screening (CKD search = 425; general population search = 86). Of the 511 articles, 315 did not contain any PSIs. PSI extraction was performed from the remaining 196 articles, with a total of 3464 PSIs identified. [Fig. 1] The full list of 3464 PSIs was filtered to identify 1775 of potential relevance to people with CKD. These PSIs were reviewed by three of the authors (FS, SH, BH) with 1231 confirmed as CKD-specific.

The CKD-specific PSIs were classified by ATC code. Three hundred seventy-one duplicates were identified and excluded. A further 19 were deemed not to fit the pre-specified criteria after discussion between reviewing authors (6 related to inpatient prescribing and 13 were deemed not to be CKD-specific). The remaining 841 unique PSIs became the final set of CKD-specific PSIs. Examples of PSIs are demonstrated in Table 1 (full list is in Additional file 2: Appendix 3).

**Table 1:** PSI examples

CKD-specific PSIs	General PSIs [not included in CKD-specific PSI library]
1. Metformin - not recommended in patients with eGFR < 45 and contraindicated in patients with eGFR < 30 [ATC code: A10BA]	1. Prescribing a traditional oral NSAID or low-dose aspirin in patients with a history of peptic ulceration without co-prescription of gastro- protection
2. Rivaroxaban - Caution in case of CrCl 15–30 ml/min - dose reduction to 15 mg/ day [ATC code: B01AF]	2. Significant drug-disease interactions in patients aged 65 and over: Congestive heart failure (systolic dysfunction) - first generation calcium channel blockers eg verapamil, diltiazem
3. Lithium - measure calcium and serum lithium levels at start of treatment; 3 monthly renal function; 6 monthly thyroid function, calcium, weight and serum lithium levels [ATC code: N05AN]	3. Management - Associated adverse therapeutic outcome in patients aged over 65: Use of theophylline without drug level monitoring at least every 6 months - theophylline toxicity
4. Drugs requiring dosage adjustment in patients with impaired kidney function and aged 65 and over: Opioids [ATC code: N02A]	4. Prescribing indicators for patients aged >65^a^: Patient with OA pain interfering with daily activities has been trialled on paracetamol (acetaminophen) 2–4 g/day
5. Medicines that may accumulate and require renal function monitoring: ARBs [ATC code: C09CA]	5. Methotrexate prescriptions should state ‘weekly’

*GFR* glomerular filtration rate, *CrCl* creatinine clearance, *NSAID* non-steroidal anti-inflammatory drug

^a^ie what patients SHOULD be taking

### CKD-specific PSIs

PSIs were found to take multiple different forms, including those that referred to single drugs (Table 1: Items 1–3), to drug classes (Item 4), and to lists of agents with particular pharmacokinetic or dynamic properties (Item 5). Some PSIs specified thresholds or ranges of kidney function (Item 1). Others referred to kidney disease non-specifically (Items 4 & 5). A variety of methods for estimating kidney function were cited, including CrCl and eGFR/GFR, though no PSI specified a method for eGFR/GFR calculation. Table 2 shows the final number of PSIs per ATC drug classification. The five ATC drug classes containing the largest proportion of CKD-specific PSIs were: Cardiovascular system (26%); Nervous system (13.4%); Blood and blood forming organs (12.4%); Alimentary and metabolism (12%); and Anti-infectives for systemic use (11.3%). Additionally, Table 2 shows the most frequent medication classes referred to in each ATC category.

**Table 2:** Final set of CKD-specific PSIs by ATC drug class and further subdivision to most frequently featured medication classes in each ATC category

ATC drug class	CKD-specific PSIs (n, %)	Top 5 medications in the ATC drug class	Number of CKD-specific PSIs (n, % of total 841 PSIs)
Cardiovascular system	219 (26.0)	Agents acting on the renin-angiotensin system	81 (9.6)
		Diuretics	48 (5.7)
		Lipid modifying agents	44 (5.2)
		Beta blocking agents	14 (1.7)
		Digitalis glycosides	11 (1.3)
Nervous system	113 (13.4)	Antidepressants	36 (4.3)
		Antiepileptics	21 (2.5)
		Opioids	19 (2.3)
		Antipsychotics	7 (0.8)
		Anxiolytics	6 (0.7)
Blood and blood forming organs	104 (12.4)	Direct factor Xa inhibitors	36 (4.3)
		Direct thrombin inhibitors	31 (3.7)
		Antithrombotic agents	9 (1.1)
		Heparins	7 (0.8)
		Other antianaemic preparations	7 (0.8)
Alimentary and metabolism	101 (12.0)	Sulfonylureas	19 (2.3)
		DPP-4 inhibitors	12 (1.4)
		GLP-1 analogues	12 (1.4)
		Biguanides	10 (1.2)
		SGLT2 inhibitors	7 (0.8)
Anti-infectives for systemic use	95 (11.3)	Other antibacterials	16 (1.9)
		Nucleoside and nucleotide reverse transcriptase inhibitors	13 (1.5)
		Beta-lactam antibacterials, penicillins	7 (0.8)
		Other beta-lactam antibacterials	7 (0.8)
		Macrolides	7 (0.8)
Antineoplastic and immunomodulating agents	81 (9.6)	Other anti-neoplastic agents	27 (3.2)
		Alkylating agents	16 (1.9)
		Anti-metabolites	14 (1.7)
		Cytotoxic antibiotics and related substances	9 (1.1)
		Plant alkaloids and other natural products	6 (0.7)
Musculo-skeletal system	68 (8.1)	Anti-inflammatory and anti-rheumatic products, non steroids	28 (3.3)
		Bisphosphonates	21 (2.5)
		Antigout preparations	14 (1.7)
		Other centrally acting agents	2 (0.2)
		Other drugs affecting bone structure and mineralisation	2 (0.2)
Antiparasitic products, insecticides and repellents	15 (1.8)	Aminoquinolones	7 (0.8)
		Biguanides	6 (0.7)
		Agents against leishmaniasis and trypanosomiasis	1 (0.1)
Genito-urinary and sex hormones	14 (1.7)	Drugs for urinary frequency/incontinence	8 (1.0)
		Drugs for erectile dysfunction	4 (0.5)
		Other urologicals	1 (0.1)
		Sex hormones	1 (0.1)
Miscellaneous	14 (1.7)	Miscellaneous	14 (1.7)
Respiratory system	6 (0.7)	Antihistamines for systemic use	5 (0.6)
		Anticholinergics	1 (0.1)
Various	6 (0.7)	Drugs for treatment of hyperkalemia and hyperphosphatemia	5 (0.6)
		Other renal system diagnostic radiopharmaceuticals	1 (0.1)
Systemic hormonal preparations	4 (0.5)	Parathyroid hormones and analogues	2 (0.2)
		Glucocorticoids	1 (0.1)
		Somatostatin and analogues	1 (0.1)
Sensory organs	1 (0.1)	Antiglaucoma preparations and miotics	1 (0.1)
Dermatologicals	0	–	0

### Patient and public involvement

Patients or the public were not involved in the design, or conduct, or reporting, or dissemination plans of our research.

## Discussion

Using a systematic search strategy and manual extraction, a library of 841 CKD-specific PSIs were identified. Indicators relating to all but one ATC drug class (dermatologicals) were found, with the majority considering cardiovascular, nervous and haematological prescribing. Drugs acting on the renin-angiotensin system, non-vitamin K oral anticoagulants, anti-diabetic medications, lipid-modifying agents, and antidepressants were particularly frequent. This library of CKD-specific PSIs is a first step towards generation of prescribing safety assessments and interventions for populations with CKD.

CKD, defined by persistently abnormal glomerular filtration rate (GFR), and/or other evidence of damage or structural abnormality, has a global prevalence approximating 13% [6, 7]. The pharmacokinetic and pharmacodynamic effects of CKD and kidney replacement therapy increase the risk of drug-related harm for the CKD population, and inappropriate prescriptions are more frequent as kidney function declines [1, 8]. Direct nephrotoxicity (e.g. from aminoglycosides and non-steroidal anti-inflammatory drugs) and idiosyncratic reactions (e.g. with metformin) add further complications [8]. The need for prescribers to consider GFR is recognised in international guidance for the management of CKD [7]. Nevertheless, reported rates of GFR-inappropriate prescriptions (according to the Summary of Product Characteristics - SPCs) range between 9 and 81% for people with CKD [1, 2].

Prescribing for people with CKD is further complicated by comorbid illness. CKD is both a cause and consequence of comorbidity, most notably microvascular, macrovascular and cardiac disease. Since the prevalence of CKD increases with age, acquisition of concurrent illness is frequent. People living with CKD may thus be recommended medications to manage causal conditions, coincidental co-morbidity, and complications of low-renal function – alongside agents which are preventative against cardiovascular disease and end stage kidney disease. Unsurprisingly, this can lead to the prescription of numerous drugs [1, 9]. The prevalence of polypharmacy – defined as the regular use of five or more medications per day – was almost 80% in a cohort of over 5000 German people with CKD [10]. Indeed, for older people with advanced CKD, the prevalence of polypharmacy is even higher (91%) and hyperpolypharmacy – defined as 10 or more medications – is common (43%) [11]. Polypharmacy increases the likelihood of inappropriate prescriptions and is associated with hospitalisation and death in people with CKD [1, 12].

An additional factor influencing renal prescribing is that conventional estimates of GFR use serum creatinine, which is affected by muscle mass and protein intake. Prescribers must be alert that age-related changes in body composition may result in lower accuracy of eGFR. KDIGO recommends prescribers use the most accurate method for GFR estimation when drug dosing [6]. Estimation of GFR using cystatin C is superior to creatinine-based approaches [13]. Cystatin-C estimation is likely to be adequate in most settings, with measurement using exogenous markers or creatinine clearance employed only where an accurate measure of GFR is required. It is noteworthy that the collated PSIs variably included eGFR, GFR and creatinine clearance, with very few recommending a method of estimation. This is likely to reflect an historical literature in an advancing field and identifies a clear area for improvement.

Clinical support systems such as computerised alerts, manual-review and eGFR-prompts have been used to reduce inappropriate prescribing in CKD populations, and can be effective in both inpatient and outpatient settings [2]. Nevertheless, these may over-estimate prescribing errors, especially of drugs for which the benefit/risk ratio may remain high even in the setting of very low GFR – for example those acting on the renin-angiotensin axis [7]. As such, to what degree ‘inappropriate’ prescriptions for people with CKD reflect calculated benefit/risk decisions versus injudicious prescribing is unclear. Expert review of individual prescriptions are likely to be difficult and costly to apply to large populations of individuals with CKD [2]. Meanwhile, simple interventions such as eGFR prompts appear ineffective and prescribing guidance can be unclear – for example, multiple eGFR calculations are used in SPCs [2]. Better approaches for identification of potential drug harm and targeting of interventions may exist. Harmonisation of product characteristics and GFR estimates with prescribing practice norms may help. Successful approaches to develop pharmacotherapy screening tools applicable to the elderly have been developed using systematically generated libraries of PSIs [14, 15]. This approach has advantages over guideline review alone, which although possible in defined patient groups (such as people with CKD) may fail to capture all important prescribing events. The work reported here aimed to assimilate PSIs specific to people with CKD as a first-step to the generation of a prescribing-safety tool for application in this group.

The main strength of this work is the use of an inclusive systematic approach before focussing on CKD-specific PSIs. This process reduces the risk of missing CKD-specific PSIs that are reported in the general literature. The main weakness is an output that requires further work before application in research or clinical settings. Removal of near-duplicate PSIs, selection of uniform dosing and GFR-estimation criteria for individual agents, appraisal in light of present evidence and supplementation with PSIs for prescribing events that did not appear is needed. Such a process will involve expert appraisal and formal approaches to consensus development beyond the scope of this work. Chronic kidney disease-specific PSIs relating to a wide range of agents were found, but the frequency of PSIs relating to particular agents should not be seen as indicative of the clinical importance of individual agents, and may simply reflect the frequency with which these agents are used, and/or general familiarity with their CKD-specific risks [1].

This work focused on outpatient prescribing for individuals with CKD, so some agents well-known to present risks in kidney disease are not captured (e.g. intravenous aminoglycosides). Inpatient prescribing differs substantially from ambulatory settings, with acute illness, acute kidney injury and daily prescription review. A separate approach for collating and categorising PSIs relevant to this setting is required. A further limitation in this study is that a single author completed full text screening of articles recommended for inclusion thus posing a risk of PSIs being missed from the literature.

## Conclusion

This study has collated a library of safety indicators relating to outpatient prescribing for people with CKD – a unique group with a higher proportion of prescribing challenges. The library generated could be used alone or alongside general PSIs to generate approaches for assessment of prescribing safety and quality for individuals and populations with CKD.

## Supplementary Information

**Additional file 1:** **Appendix 1** - Systematic literature search terms. **Appendix 2** - CKD-specific PSI selection.

**Additional file 2: Appendix 3 - **841 CKD-specific PSIs.

## Data Availability

All data generated during this study are included in this published article (and its supplementary information files). This data can be used to assess the safety and quality of prescribing within a cohort of people with chronic kidney disease.

## Abbreviations

CKD: Chronic kidney disease
PSIs: Prescribing safety indicators
ATC: Anatomical Therapeutic Chemical
GFR: Glomerular filtration rate
SPC: Summary of Product Characteristics
