# Supplementary material for: A systematically collated library of prescribing safety indicators for people with chronic kidney disease
Source: BMC Nephrol. 2020 Nov 18;21:493. doi: 10.1186/s12882-020-02158-0 (PMC7672989; doi:10.1186/s12882-020-02158-0)
Supplement: Supplementary file 1 — Additional file 1: Appendix 1 - Systematic literature search terms. Appendix 2 - CKD-specific PSI selection. [file 12882_2020_2158_MOESM1_ESM.docx]

**Additional file 1 – Systematic literature search terms**

| **Clinical Setting** | **Prescribing** | **Types of Tools** |
| --- | --- | --- |
| Family physician  Primary care  Family practice  General practice  Ambulatory care  Ambulatory health  Ambulatory health-care  Ambulatory healthcare  Community health  Community health-care  Community healthcare  Primary health  Primary health-care  Primary healthcare  Primary physician  Generalist  Family medicine | Administration error  Administration errors  Dispensing error  Dispensing errors  Medication error  Medication errors  Medical mistake  Medical mistakes  Prescription error  Prescription errors  Prescribing error  Prescribing errors  Prescribing fault  Prescribing faults  Medical error  Medical errors  Adverse event  Adverse events  Adverse effect  Adverse effects  Adverse reaction  Adverse reactions  Harm  Harms  Malpractice  Safety  Safety-culture | Scale  Scales  Survey  Surveys  Questionnaire  Questionnaires  Instrument  Instruments  Indicator  Indicators  Outcome assessment  Outcome assessments  Patient reported outcome  Patient reported outcomes  Patient experience  Patient experiences  Practice guideline  Practice guidelines  Tool  Tools  Toolkit  Toolkits  Quality assurance |

Table 3: Search terms used for general literature search

*General literature search terms:* (family physician.tw or primary care.tw or family practice.tw or general practice.tw or ambulatory care.tw or ambulatory health.tw or ambulatory health-care.tw or ambulatory healthcare.tw or community health.tw or community health-care.tw or community healthcare.tw or primary health.tw or primary health-care.tw or primary healthcare.tw or primary physician.tw or generalist.tw or family medicine.tw) AND (administration error.tw or administration errors.tw or dispensing error.tw or dispensing errors.tw or medication error.tw or medication errors.tw or medical mistake.tw or medical mistakes.tw or prescription error.tw or prescription errors.tw or prescribing error.tw or prescribing errors.tw or prescribing fault.tw or prescribing faults.tw or medical error.tw or medical errors.tw or adverse event.tw or adverse events.tw or adverse effect.tw or adverse effects.tw or adverse reaction.tw or adverse reactions.tw or harm.tw or harms.tw or malpractice.tw or safety.tw or safety-culture.tw) AND (scale.tw or scales.tw or survey.tw or surveys.tw or questionnaire.tw or questionnaires.tw or instrument.tw or instruments.tw or indicator.tw or indicators.tw or outcome assessment.tw or outcome assessments.tw or patient reported outcome.tw or patient reported outcomes.tw or patient experience.tw or patient experiences.tw or practice guideline.tw or practice guidelines.tw or tool.tw or tools.tw or toolkit.tw or toolkits.tw or quality assurance.tw)

|  | **Clinical setting** | **Prescribing** | **Types of tools** | **Chronic kidney disease** |
| --- | --- | --- | --- | --- |
| MEsH |  | Drug prescriptions  Drug therapy  Inappropriate prescribing  Medication errors  Potentially inappropriate medication list  Drug interactions | Quality indicators, Health Care | Renal insufficiency |
| Wordsearch  (.tw) | Family physician  Primary care  Family practice  General practice  Ambulatory care  Ambulatory health  Ambulatory health-care  Ambulatory healthcare  Community health  Community health-care  Community healthcare  Primary health  Primary health-care  Primary healthcare  Primary physician  Generalist  Family medicine  Clinic  Outpatient  Out-patient | Administration error*  Dispensing error*  Medication error*  Prescription error*  Prescribing error*  Prescribing fault*  Medical error*  Medical mistake*  Adverse event*  Adverse effect*  Adverse react*  Drug interact*  Drug-drug interact*  DDI*  Therapeutic drug monitoring  Harm*  Safety* | Scale*  Survey*  Questionnaire*  Instrument*  Indicator*  Outcome assessment*  Patient reported outcome*  Patient experience*  Practice guideline*  Tool*  Quality assurance  Checklist*  Check-list*  Score*  Recommendation*  Warning system*  Alert system*  Flag*  Trigger alert*  Best practice* | CKD  Chronic kidney disease  Chronic renal disease  Chronic kidney failure  Chronic renal failure  Chronic kidney insufficiency  Chronic renal insufficiency  End stage kidney disease  End stage renal disease  Nephrotoxicity  eGFR < 90*  eGFR<90*  eGFR <90*  eGFR less than 90*  eGFR below 90*  GFR < 90*  GFR<90*  GFR <90*  GFR less than 90*  GFR below 90*  Glomerular filtration rate < 90*  Glomerular filtration rate<90*  Glomerular filtration rate <90*  Glomerular filtration rate less than 90*  Glomerular filtration rate below 90*  eGFR < 60*  eGFR<60*  eGFR <60*  eGFR less than 60*  eGFR below 60*  GFR < 60*  GFR<60*  GFR <60*  GFR less than 60*  GFR below 60*  Glomerular filtration rate < 60*  Glomerular filtration rate<60*  Glomerular filtration rate <60*  Glomerular filtration rate less than 60*  Glomerular filtration rate below 60*  eGFR < 30*  eGFR<30*  eGFR <30*  eGFR less than 30*  eGFR below 30*  GFR < 30*  GFR<30*  GFR <30*  GFR less than 30*  GFR below 30*  Glomerular filtration rate < 30*  Glomerular filtration rate<30*  Glomerular filtration rate <30*  Glomerular filtration rate less than 30*  Glomerular filtration rate below 30*  eGFR < 25*  eGFR<25*  eGFR <25*  eGFR less than 25*  eGFR below 25*  GFR < 25*  GFR<25*  GFR <25*  GFR less than 25*  GFR below 25*  Glomerular filtration rate < 25*  Glomerular filtration rate<25*  Glomerular filtration rate <25*  Glomerular filtration rate less than 25*  Glomerular filtration rate below 25*  eGFR < 20*  eGFR<20*  eGFR <20*  eGFR less than 20*  eGFR below 20*  GFR < 20*  GFR<20*  GFR <20*  GFR less than 20*  GFR below 20*  Glomerular filtration rate < 20*  Glomerular filtration rate<20*  Glomerular filtration rate <20*  Glomerular filtration rate less than 20*  Glomerular filtration rate below 20*  eGFR < 15*  eGFR<15*  eGFR <15*  eGFR less than 15*  eGFR below 15*  GFR < 15*  GFR<15*  GFR <15*  GFR less than 15*  GFR below 15*  Glomerular filtration rate < 15*  Glomerular filtration rate<15*  Glomerular filtration rate <15*  Glomerular filtration rate less than 15*  Glomerular filtration rate below 15*  eGFR < 10*  eGFR<10*  eGFR <10*  eGFR less than 10*  eGFR below 10*  GFR < 10*  GFR<10*  GFR <10*  GFR less than 10*  GFR below 10*  Glomerular filtration rate < 10*  Glomerular filtration rate<10*  Glomerular filtration rate <10*  Glomerular filtration rate less than 10*  Glomerular filtration rate below 10*  eGFR < 5*  eGFR<5*  eGFR <5*  eGFR less than 5*  eGFR below 5*  GFR < 5*  GFR<5*  GFR <5*  GFR less than 5*  GFR below 5*  Glomerular filtration rate < 5*  Glomerular filtration rate<5*  Glomerular filtration rate <5*  Glomerular filtration rate less than 5*  Glomerular filtration rate below 5*  Creatinine clearance*.tw  CrCl*.tw |

Table 4: Search terms used for CKD-specific literature search

*CKD-specific search terms*: (family physician.tw or primary care.tw or family practice.tw or general practice.tw or ambulatory care.tw or ambulatory health.tw or ambulatory health-care.tw or ambulatory healthcare.tw or community health.tw or community health-care.tw or community healthcare.tw or primary health.tw or primary health-care.tw or primary healthcare.tw or primary physician.tw or generalist.tw or family medicine.tw or clinic*.tw or outpatient*.tw or out-patient*.tw) AND (exp Drug Prescriptions/ or exp Drug Therapy/ or exp Inappropriate Prescribing/ or exp Medication Errors/ or exp Potentially Inappropriate Medication List/ or exp drug interactions/ or administration error*.tw or dispensing error*.tw or medication error*.tw or prescription error*.tw or prescribing error*.tw or prescribing fault*.tw or medical error*.tw or medical mistake*.tw or adverse event*.tw or adverse effect*.tw or adverse react*.tw or drug interact*.tw or drug-drug interact.tw or DDI*.tw or therapeutic drug monitoring.tw or harm*.tw or safety*.tw) AND (exp Quality Indicators, Health Care/ or scale*.tw or survey*.tw or questionnaire*.tw or instrument*.tw or indicator*.tw or outcome assessment*.tw or patient reported outcome*.tw or patient experience*.tw or practice guideline*.tw or tool*.tw or quality assurance.tw or checklist*.tw or check-list*.tw or score*.tw or recommendation*.tw or warning system*.tw or alert system*.tw or flag*.tw or trigger alert*.tw or best practice*.tw) AND (exp Renal Insufficiency/ or CKD.tw or chronic kidney disease.tw or chronic renal disease.tw or chronic kidney failure.tw or chronic renal failure.tw or chronic kidney insufficiency.tw or chronic renal insufficiency.tw or end stage kidney disease.tw or end stage renal disease.tw or nephroxicity.tw or eGFR < 90*.tw or eGFR<90*.tw or eGFR <90*.tw or eGFR less than 90*.tw or eGFR below 90*.tw or GFR < 90*.tw or GFR<90*.tw or GFR <90*.tw or GFR less than 90*.tw or GFR below 90*.tw or glomerular filtration rate < 90*.tw or glomerular filtration rate<90*.tw or glomerular filtration rate <90*.tw or glomerular filtration rate less than 90*.tw or glomerular filtration rate below 90*.tw or eGFR < 60*.tw or eGFR<60*.tw or eGFR <60*.tw or eGFR less than 60*.tw or eGFR below 60*.tw or GFR < 60*.tw or GFR<60*.tw or GFR <60*.tw or GFR less than 60*.tw or GFR below 60*.tw or glomerular filtration rate < 60*.tw or glomerular filtration rate<60*.tw or glomerular filtration rate <60*.tw or glomerular filtration rate less than 60*.tw or glomerular filtration rate below 60*.tw or eGFR < 30*.tw or eGFR<30*.tw or eGFR <30*.tw or eGFR less than 30*.tw or eGFR below 30*.tw or GFR < 30*.tw or GFR<30*.tw or GFR <30*.tw or GFR less than 30*.tw or GFR below 30*.tw or glomerular filtration rate < 30*.tw or glomerular filtration rate<30*.tw or glomerular filtration rate <30*.tw or glomerular filtration rate less than 30*.tw or glomerular filtration rate below 30*.tw or eGFR < 25*.tw or eGFR<25*.tw or eGFR <25*.tw or eGFR less than 25*.tw or eGFR below 25*.tw or GFR < 25*.tw or GFR<25*.tw or GFR <25*.tw or GFR less than 25*.tw or GFR below 25*.tw or glomerular filtration rate < 25*.tw or glomerular filtration rate<25*.tw or glomerular filtration rate <25*.tw or glomerular filtration rate less than 25*.tw or glomerular filtration rate below 25*.tw or eGFR < 20*.tw or eGFR<20*.tw or eGFR <20*.tw or eGFR less than 20*.tw or eGFR below 20*.tw or GFR < 20*.tw or GFR<20*.tw or GFR <20*.tw or GFR less than 20*.tw or GFR below 20*.tw or glomerular filtration rate < 20*.tw or glomerular filtration rate<20*.tw or glomerular filtration rate <20*.tw or glomerular filtration rate less than 20*.tw or glomerular filtration rate below 20*.tw or eGFR < 15*.tw or eGFR<15*.tw or eGFR <15*.tw or eGFR less than 15*.tw or eGFR below 15*.tw or GFR < 15*.tw or GFR<15*.tw or GFR <15*.tw or GFR less than 15*.tw or GFR below 15*.tw or glomerular filtration rate < 15*.tw or glomerular filtration rate<15*.tw or glomerular filtration rate <15*.tw or glomerular filtration rate less than 15*.tw or glomerular filtration rate below 15*.tw or eGFR < 10*.tw or eGFR<10*.tw or eGFR <10*.tw or eGFR less than 10*.tw or eGFR below 10*.tw or GFR < 10*.tw or GFR<10*.tw or GFR <10*.tw or GFR less than 10*.tw or GFR below 10*.tw or glomerular filtration rate < 10*.tw or glomerular filtration rate<10*.tw or glomerular filtration rate <10*.tw or glomerular filtration rate less than 10*.tw or glomerular filtration rate below 10*.tw or eGFR < 5*.tw or eGFR<5*.tw or eGFR <5*.tw or eGFR less than 5*.tw or eGFR below 5*.tw or GFR < 5*.tw or GFR<5*.tw or GFR <5*.tw or GFR less than 5*.tw or GFR below 5*.tw or glomerular filtration rate < 5*.tw or glomerular filtration rate<5*.tw or glomerular filtration rate <5*.tw or glomerular filtration rate less than 5*.tw or glomerular filtration rate below 5*.tw or Creatinine clearance*.tw or CrCl*.tw)

**Additional file 1: APPENDIX 2 – CKD-specific PSI selection**

| **CKD-specific PSI filter terms** |
| --- |
| CKD  Kidney  Renal  End stage  Endstage  GFR  ESKD  ESRD  ESKF  ESRF  Glom  Creatinine  CrCl  AKI  ARF  Nephro*  Scr  U&E  U+E  Potassium  K  K+  [K]  [K+]  Urea  Uria  Risk |

Table 5: CKD-specific PSI filter terms
